# Self‐Enhancing Gel Polymer Electrolyte by In Situ Construction for Enabling Safe Lithium Metal Battery

**DOI:** 10.1002/advs.202103663

**Published:** 2021-12-11

**Authors:** Dongli Chen, Ming Zhu, Peibin Kang, Tao Zhu, Haocheng Yuan, Jinle Lan, Xiaoping Yang, Gang Sui

**Affiliations:** ^1^ State Key Laboratory of Organic–Inorganic Composites College of Materials Science and Engineering Beijing University of Chemical Technology Beijing 100029 China; ^2^ Shanghai Institute of Space Power‐Sources Shanghai 200245 China

**Keywords:** electrochemical performance, in situ polymerization, lithium metal batteries, poly‐1,3‐dioxolane, polymer electrolyte

## Abstract

Lithium metal battery (LMB) possessing a high theoretical capacity is a promising candidate of advanced energy storage devices. However, its safety and stability are challenged by lithium dendrites and the leakage of liquid electrolyte. Here, a self‐enhancing gel polymer electrolyte (GPE) is created by in situ polymerizing 1,3‐dioxolane (DOL) in the nanofibrous skeleton for enabling safe LMB. The nanofiber membrane possesses a better affinity with poly‐DOL (PDOL) than commercial separator for constructing homogeneous GPE with enhanced ion conductivity. Furthermore, polydopamine is introduced on nanofiber membrane to form hydrogen bonding with PDOL and bis((trifluoromethyl)sulfonyl)imide anion, dramatically improving the mechanical strength, ionic conductivity, and transference number of GPE. Besides, molecular dynamic simulation is used to reveal the intrinsic factors of high ionic conductivity and reinforcing effect in the meantime. Consequently, the LiFePO_4_//Li batteries using self‐enhancing GPE show extraordinary cyclic stability over 800 cycles under high current density of 2 C, with a capacity decay of 0.021% per cycle, effectively suppressing the growth of lithium dendrites. This ingenious strategy is expected to manufacture advanced performance and high safety LMBs and compatible with the current battery production.

## Graphical abstract

A self‐enhancing gel polymer electrolyte (GPE) based on in situ construction display favorable mechanical strength (10.1 MPa), high ionic conductivity (2.39 × 10^−3^ S cm^−1^), and Li^+^ transference number (0.59). The GPE shows extraordinary cyclic stability over 800 cycles at 2 C and effective suppression of Li dendrites.

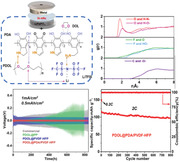

## 1 Introduction

In pursuit of higher energy density, the researchers have paid a lot of attention to lithium metal battery (LMB) due to its high theoretical specific capacity (3860 mAh g^−1^) and lowest oxidation‐reduction potential (−3.04 V vs Li^+^/Li).^[^ ^1^ ^]^ However, there are still many problems to be tackled, such as the flammability and easy leakage of liquid electrolyte (LE), safety hazards caused by lithium dendrites and unsatisfied cycle stability.^[^ ^2^ ^]^ To overcome these issues, the safe polymer electrolyte is a promising candidate.^[^ ^3^ ^]^

In general, the polymer electrolytes can be divided into solid polymer electrolytes (SPEs) and gel polymer electrolytes (GPEs) according to their composition. Although SPEs can mitigate the growth of Li dendrites and improve the safety of batteries, they often display poor interfacial compatibility and low room‐temperature lithium ion (Li^+^) conductivity, not meeting the practical demands of batteries.^[^ ^4^ ^]^ By contrast, the GPEs are applicable to develop safe and high‐performance LMB because the GPEs possess high ionic conductivity and better interface contact with Li metal at room temperature.^[^ ^5^ ^]^ However, the GPEs show poor mechanical strength, and their thickness is often hundreds of microns for assembling.^[^ ^6^ ^]^ For example, Liu et al. reported a cellulose based GPE with a high ionic conductivity of 8.9 × 10^−3^ S cm^−1^ at room temperature, but the GPE displayed a tensile strength of only 0.14 MPa even though its thickness was increased.^[^ ^6a^ ^]^ A supple cross‐linked GPE was designed by Tsao and co‐workers, and the GPE possessed high LE uptake and ionic conductivity of 2.0 × 10^−3^ S cm^−1^ at 30 °C.^[^ ^7^ ^]^ However, the thickness of the GPE was over 150 µm for maintaining mechanical properties of the membrane after gelation. Similarly, Su et al. constructed a novel semi‐interpenetrating ion gel electrolyte, while the thick glass fiber as skeleton materials increased the transfer distance of Li^+^ and the battery polarization, leading to reduced energy density.^[^ ^6d^ ^]^ Besides, to better suppress the lithium dendrites, the GPEs are modified and functionalized with inorganic nanoparticles.^[^ ^8^ ^]^ Nevertheless, the fabrication of the GPE with functionality was complicated and difficult for large‐scale application, and the final GPE showed deteriorated interface compatibility with cathode and anode.^[^ ^9^ ^]^ Xu et al. introduced multifunctional rGO (rGO‐PEG‐NH_2_) into the polyvinylidene fluoride‐hexafluoropropylene (PVDF‐HFP) based GPE for inhibiting the growth of Li dendrites, but the synthesis of rGO‐PEG‐NH_2_ was complicated.^[^ ^10^ ^]^ Mg(II)‐based metal‐organic‐framework material was chosen to modify PVDF‐based GPE for preventing Li dendrites and immobilizing anions, which is difficult to large‐scale production.^[^ ^11^ ^]^ A core–shell structured SiO_2_ nanoparticles as fillers was incorporated into GPE for suppressing Li dendrites. However, the unsatisfactory interface compatibility with electrodes resulted in a high interface impedance of over thousands and limited long‐cycle performance.^[^ ^9b^ ^]^ Therefore, it was urgently needed to simply and trustworthily create a thin GPE with good strength and interface compatibility in the general commercialization of LMBs.

Recently, the novel in situ polymerization method has been used to build fast Li^+^ interfacial transport in polymer electrolyte, and the simple routes can directly be applied in the battery industries.^[^ ^12^ ^]^ The precursor electrolyte with low viscosity can penetrate into the entire region of the cell, and the resultant in situ formed GPE can achieve a decreased interfacial resistance, which was smaller than that of cell with premanufactured polymer electrolyte.^[^ ^12^, ^13^ ^]^ The 1, 3‐dioxolane (DOL), a common LE solvent, can form polymer matrix for GPE via ring‐opening polymerization.^[^ ^13^, ^14^ ^]^ Currently, some works have been reported that in situ prepared poly‐DOL electrolyte (PDOL) can significantly enhance interface compatibility in LMBs.^[^ ^13^, ^15^ ^]^ For instance, Liu and colleagues proposed a safe PDOL GPE with double‐salt system (LiDFOB‐LiTFSI) and succinonitrile as plasticizer, while the addition of succinonitrile reduced the strength of GPE and receded suppression of Li dendrites.^[^ ^15^ ^]^ Ma and co‐workers introduced the in situ polymerization of DOL to common carbonate‐based electrolyte forming a hybrid solid/liquid electrolyte, but the residual LE in the hybrid electrolyte had the risk of leakage and impaired the mechanical properties of GPE, leading to a greatly weakened ability of inhibiting Li dendrites.^[^ ^16^ ^]^ The GPE based on the trioxymethylene in situ copolymerized with DOL was reported by Liu et al., and the GPE exhibited good interface compatibility.^[^ ^17^ ^]^ However, the conductivity of GPE was sacrificed with the poor compatibility between PP and PDOL, and the porosity of PP and low strength of PDOL were not able to inhibit the growth of Li dendrites.

Herein, a self‐enhancing thin GPE was in situ constructed by polymerizing DOL in the nanofibrous skeleton for enabling safe gel LMB. The skeleton was consisted of nanofiber membrane of polydopamine‐modified PVDF‐HFP (PDA/PVDF‐HFP), which possessed a good affinity with PDOL and DOL for enhancing ion conductivity. The DOL precursor solution can be firmly absorbed in the porous membrane to swell PVDF‐HFP chains well. After polymerization, the GPE without organic liquid residue ensured sufficient safety of the battery. Furthermore, the membrane skeleton modified by PDA can form hydrogen bonds with PDOL, dramatically improving the mechanical strength of GPE and achieving effective inhibition of Li dendrites. Consequently, the novel GPE exhibited an excellent mechanical strength and a desired ionic conductivity. The batteries assembled with self‐enhancing GPE showed exceptional cyclic stability under high current density, owing to suppression of Li dendrites. This work broadens the prospects for the preparation and application of high‐performance GPE, which is compatible with the current battery production processes.

## 2 Results and Discussion

### 2.1 Characterization of PDOL, Nanofiber Membranes, and As‐Prepared GPEs

**Figure 1:**
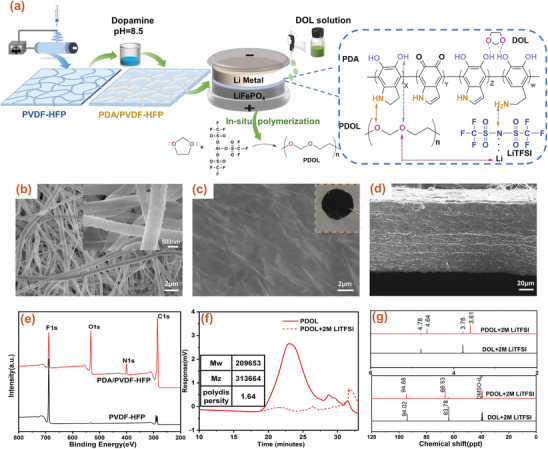
a) Preparation of 3D PDOL@PDA/PVDF‐HFP gel polymer electrolyte via in situ polymerization. SEM images of PDA/PVDF‐HFP membrane and PDOL@PDA/PVDF‐HFP GPE. b,c) Surface and d) Cross section. (The inset was optical image of PDOL@PDA/PVDF‐HFP GPE). e) XPS spectra of PVDF‐HFP and PDA/PVDF‐HFP membranes. f) The GPC profile for PDOL via in situ polymerization. g) ^1^H and ^13^C NMR spectra of PDOL and DOL.

An innovative route was provided to prepare self‐enhancing GPE based on PDOL via in situ polymerization, and the details were illustrated in **Figure** **1**a. During the fabrication, the precursor (the details of precursor were in experimental part of the Supporting Information) was injected into the PDA/PVDF‐HFP electrospinning membrane and the assembled batteries were stood for 3 days for complete polymerization. As‐prepared GPEs were nonflowable, which was demonstrated by polymerization in the bottle in Figure S1 (Supporting Information), ensuring the safety of batteries. To improve the strength of GPE, the nanofiber membranes were modified with PDA due to the hydrogen bond interactions between PDOL and PDA. The functional groups (e.g., —NH— and —OH) in PDA formed cross‐linking networks with polar oxygen atom (O) of PDOL molecular through hydrogen bonding (dotted line in Figure 1a), which improved the mechanical properties of GPE. Notably, this fabrication route was compatible with the current manufacture process of commercial batteries, and effectively simplified the complicated preparation process of GPEs.

In order to further analyze the physical properties and chemical structure of GPEs, the scanning electron microscope (SEM), X‐ray photoelectron spectroscopy (XPS), and nuclear magnetic resonance (NMR) tests were carried out. In Figure 1b, no agglomerated PDA particles existed in PDA/PVDF‐HFP membrane and the fibers was rough and uniform, which manifested that PDA grew evenly on the PVDF‐HFP nanofibers, rather than agglomerating to block the holes of nanofiber membrane. Besides, the surfaces of GPEs were smooth and dense, and no obvious pores can be found after PDOL integrated with membranes shown in Figure 1c,d; and Figure S2 (Supporting Information), demonstrating that PDOL was evenly filled into the nanofiber membranes, and PDOL interwoven networks were formed. Additionally, the insets were optical photos of PDOL@PDA/PVDF‐HFP, PDOL@PVDF‐HFP, PDOL@PP GPEs in Figure 1c; and Figure S2 (Supporting Information). Clearly, PDOL@PDA/PVDF‐HFP GPE was dark brown and erected without shrinkage, indicating that it was self‐supporting. The final thickness of GPE was about 90 µm.

**Figure 2:**
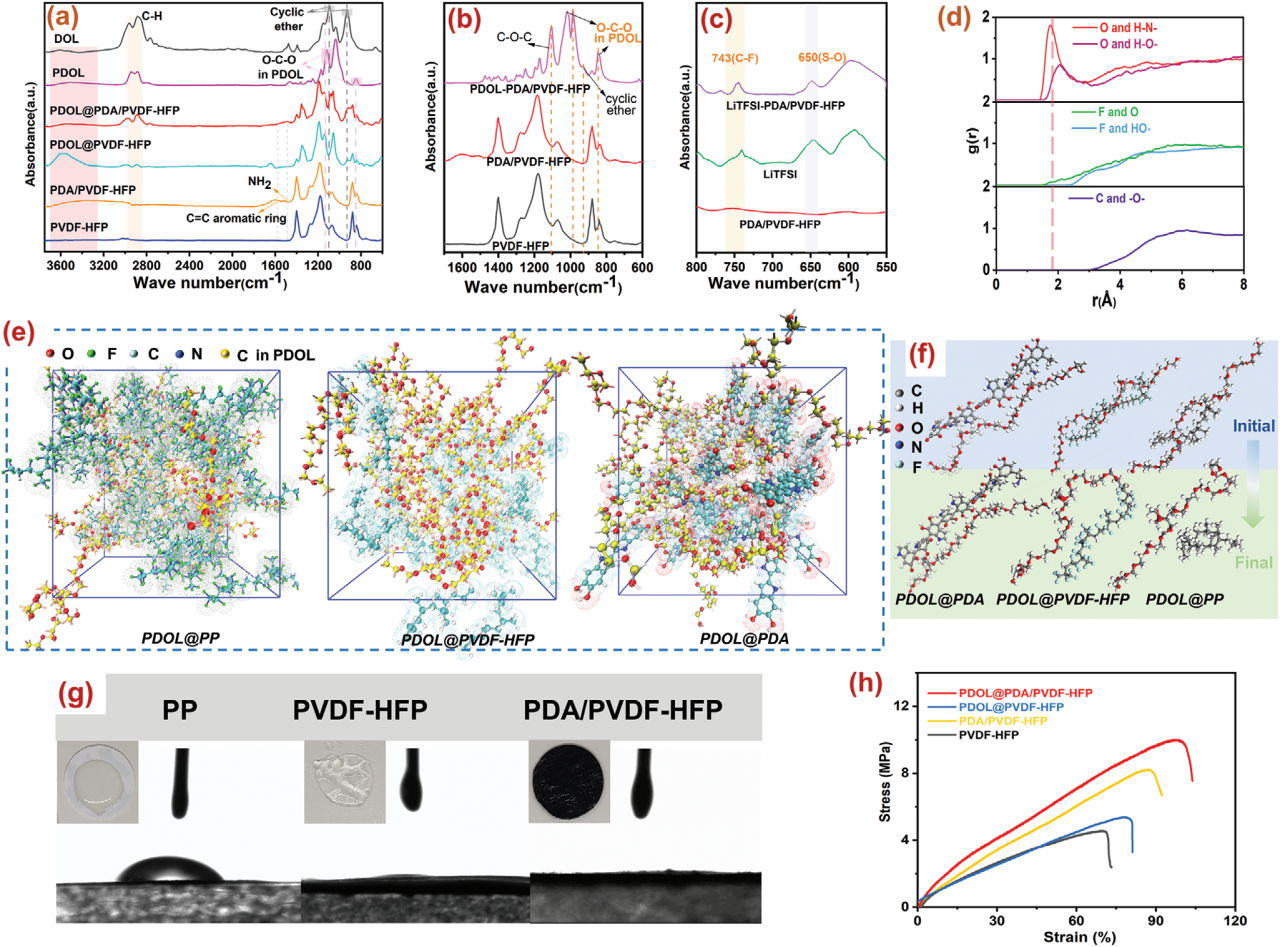
a) FTIR spectroscopy of DOL, PDOL, PVDF‐HFP, and PDA/PVDF‐HFP membranes, PDOL@PVDF‐HFP and PDOL@PDA/PVDF‐HFP GPEs and b,c) Their partial enlarged diagrams. d) Radial distribution functions g(r) of O atoms in PDOL with H atoms of —NH— and —OH groups in PDA, or F atoms in PVDF‐HFP or C atoms in PP, respectively. e) Snapshots obtained from MD simulations of PDOL and PDA, PP, PVDF‐HFP. f) Schematic diagram of the relative position changes between PDOL chains and chains of different membranes during the movement of chains. g) Contact angles on three kinds of membranes. (The insets were photographs of these three membranes after absorbing polymeric precursors after 3 s). h) Stress–strain curves of PVDF‐HFP and PDA/PVDF‐HFP membranes, PDOL@PVDF‐HFP and PDOL@PDA/PVDF‐HFP GPEs.

The XPS (Figure 1e; and Figure S3, Supporting Information) and Fourier transform infrared spectroscopy (FTIR) (**Figure** **2**a) spectra also demonstrated the even growth of PDA outside nanofibers. Clearly, two strong new peaks (O1s, 533.0 eV and N1s, 400.1 eV) appeared after the introduction of PDA in XPS spectrum. Especially in N1s spectrum (Figure S3a, Supporting Information), the peaks at 398.0, 400.0, and 401.9 eV indicated the existence of pyridine N, pyrrolidine N and graphite N, respectively, consistent with the N‐species in PDA.^[^ ^18^ ^]^ Meanwhile, the weakening of F1s peak was the result of PDA growth. It also caused a slight decrease in the porosity (Figure S5a, Supporting Information). Compared with PP separator, electrospin nanofiber membranes had larger holes and higher porosity which were consistent with SEM results. Furthermore, it was obtained that the molecular weight and dispersion coefficient of PDOL were around 313 664 (Mz) and 1.64, respectively, via gel permeation chromatography (GPC) measurement (Figure 1f). Besides, ^1^H NMR and ^13^C NMR measurements (Figure 1g) were conducted to further certify the polymerization reaction of DOL monomer. The chemical shifts at 3.78 and 4.78 ppm corresponded to —CH_2_—CH_2_—O— and —CH_2_—O—CH_2_— on the cyclic ether monomer, respectively. After polymerization, the above chemical shifts were moved to 3.61 ppm belonging to the H on the —O—CH_2_—CH_2_—O— group and 4.64 ppm assigned to —O—CH_2_—O—, respectively. Meanwhile, the carbon chemical shifts assigned to main chain were shifted from 94.82 to 95.4 ppm and from 64.3 to 66.7 ppm, respectively. The results of ^1^H NMR and ^13^C NMR spectra were accordant with the theory and related literature, indicating the successful polymerization of DOL.^[^ ^13^, ^19^ ^]^ Furthermore, no obvious peaks related to the crystallization or melting behavior were observed in the differential scanning calorimetry curves (−20–60 °C) (Figure S5b, Supporting Information), which meant that the polymer electrolyte was almost amorphous due to the plasticizing effects of LiTFSI, favoring the Li^+^ conductivity.^[^ ^20^ ^]^

### 2.2 Analysis of Hydrogen Bond Interactions in As‐Prepared GPEs

PDA coating and the polymerization of DOL in the 3D framework were confirmed by ATR‐FTIR spectroscopy (3800–600 cm^−1^) in Figure 2a,b. Compared with the pristine membrane, new peaks appeared in FTIR after the introduction of PDA: the broad peak during 3660 and 3107 cm^−1^ represented N—H/O—H stretching vibration, what's more, the peaks at 1487 and 1574 cm^−1^ ascribed to N—H bending vibration and the overlap of the C═C resonance vibration in the aromatic ring (Figure 2a; and Figure S5e, Supporting Information). From another perspective, the results also embraced that DOL was successfully cationic polymerized into PDOL. Apparently, the characteristic peaks of the cyclic ether in DOL (925, 1096 cm^−1^) disappeared significantly after polymerization,^[^ ^21^ ^]^ and the characteristic peak of —O—C—O chains at 845 cm^−1^ in PDOL appeared suddenly, indicating the successful polymerization.^[^ ^21^ ^]^ Due to the hydrogen‐bond interaction between PDOL and PDA molecules (Figure 1a), the interaction between PDA molecules was weakened, resulting in a smaller and narrower of the —OH/—NH broad peak.^[^ ^22^ ^]^ (Figure 2a). Similarly, PDA can also interact with TFSI^−1^ anions to facilitate the dissolution of lithium salts. Hence, the characteristic peaks at 743, 650, and 1630 cm^−1^ related to the C—F, S—O groups and LiTFSI aggregation, respectively, (Figure 2c; and Figure S5d, Supporting Information) significantly decreased after PDA introduced.^[^ ^6c^ ^]^ Therefore, the GPE was hopeful to achieve a higher conductivity and Li^+^ transference number (*t* ^+^). Molecular dynamics (MD) simulations further revealed the interaction between PDA and PDOL. In Figure 2d, no obvious peaks appeared in radial distribution functions (RDFs) for O atoms of PDOL with H atoms in PVDF‐HFP or PP. On the contrary, RDFs between O atoms and H atoms in PDA (from —NH— and —OH groups) showed two distinct peaks at 1.7 and 2 Å, respectively, indicating the higher possibility of finding H atoms from PDA around O atom in PDOL than that in PVDF‐HFP and PP systems, reflecting the formation of intermolecular hydrogen bonds in the PDA/PVDF‐HFP system.^[^ ^23^ ^]^ This was also consistent with the results of FTIR. And explained the better mechanical properties of PDOL@PDA/PVDF‐HFP GPE than the others. The corresponding snapshots obtained from MD simulations of PDOL and PDA were shown in Figure 2e. Schematic diagram described (Figure 2f) that the effect of the interaction between different molecular chains on the chain movement, indicating a strong interaction between the polar groups in PDA and PDOL chains.

The contribution of PDA on the wettability of membrane was also confirmed by the contact angle measurement as shown in Figure 2g. It was easy to figure out that the average contact angles of PP, PVDF‐HFP, and PDA/PVDF‐HFP membranes were 50°, 4°, and 0°, respectively, which implied a better wettability of PDA/PVDF‐HFP nanofiber membrane. Meanwhile, the wetting behavior and self‐supporting performance were assessed by dropping 30 µL of polymeric precursors on the membranes. PDA coating was conducive to the wettability between precursors and backbone due to abundant polar functional groups in PDA. From the optical photographs in Figure 2g, intuitively, the precursors spread out rapidly on the PVDF‐HFP and PDA/PVDF‐HFP membranes which also had the larger diffusion area, meaning the better wettability and affinity. This was on account of multiply tortuous pores and forest‐like electrospinning membrane formed by rough nanofibers, which enhanced the ability to trap the precursor, allowing more Li^+^ transport freely inside the 3D composite electrolyte. However, the PVDF‐HFP membrane tended to shrinkage and had no self‐supporting quality after absorbing precursors. On the contrary, PDA/PVDF‐HFP membrane suffered no changes, implying a certain role of PDA for improving mechanical properties. Besides, the precursor accumulated into a spherical shape on the PP membrane due to nonpolarity, which was not conducive to rapid infiltration of the precursors.

The mechanical properties of GPE play a significant role in its practical application, it should be enough to withstand the tension during the battery manufacturing process, and good mechanical strength can effectively prevent lithium dendrites. The tensile strengths of original membranes and PDOL‐based GPEs were detected in Figure 2h. It was found that the mechanical strength of PDA/PVDF‐HFP membrane was distinctly enhanced from 4.6 to 8.2 MPa after PDA coated, which was attributed that the hydrogen bond interaction between PDA provided physical cross‐linking among the forest‐like PVDF‐HFP nanofibers.^[^ ^18^, ^24^ ^]^ Moreover, with the integration of PDOL, the tensile strength of GPEs was distinctly improved further. Among them, the tensile strength of PDOL@PDA/PVDF‐HFP GPE reached 10.1 MPa, much higher than other GPEs, fully meeting the requirements of production process and practical application. Meanwhile, the elongation when breaking increased by around 27% than PDOL@PVDF‐HFP. The fine mechanical properties were due to both chemical and physical factors. The PDA on the membrane was chemically interacted with PDOL. Additionally, the DOL polymerized and uniformly filled the pores of the electrospinning membranes. The PDOL interweaving with nanofibers provided physical crosslinking.^[^ ^25^ ^]^ It was confirmed by SEM images (Figure 1) and supported by MD simulations results (Figure 2).

### 2.3 Electrochemical Performance of PDOL‐Based GPEs

**Figure 3:**
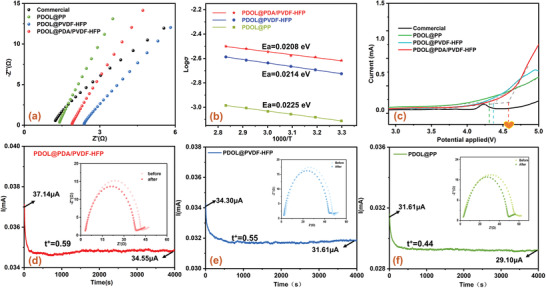
The basic electrochemical performance of PDOL@PP, PDOL@PVDF‐HFP, and PDOL@PDA/PVDF‐HFP cells. a) Nyquist plots, b) Arrhenius plots, c) Linear sweep voltammetry (LSV) curves and d–f) Chronoamperometry curves with a step voltage of 2 mV (The insets displayed EIS plot before and after polarization) of cells with PDOL@PP, PDOL@PVDF‐HFP, and PDOL@PDA/PVDF‐HFP.

In the Nyquist plots (Figure S6a–c, Supporting Information), the bulk resistance (*R*_b_) increased over time, corresponding to the decrease in conductivity, while the resistance remains almost unchanged after 3 days, indicating that the polymerization was completed and the material performance had reached a stable state. The conductivities of PDOL@PP, PDOL@PVDF‐HFP, PDOL@PDA/PVDF‐HFP were 0.9 × 10^−3^, 1.88 × 10^−3^, and 2.39 × 10^−3^ S cm^−1^ at 25 °C, respectively (**Figure** **3**a), calculated by Equation (S2) (Supporting Information), which were comparable to that of the up‐to‐date GPEs. That was due to the fact that the concentration of free Li^+^ was significantly increased in the interface region between PDA and PDOL from MD simulation (Figure S7, Supporting Information), which suggested that the introduction of PDA facilitated the dissolution of Li^+^, thereby increasing the ionic conductivity. This is consistent with the results of Nyquist plots (Figure 3a) and FTIR (Figure 2c).

Moreover, the temperature dependence of the Li^+^ conductivity was presented in Figure 3b. With the Arrhenius equation in Equation S3 (Supporting Information), we obtained the oblique lines passing through the dates point, and the measured conductivity was well fitted by Arrhenius model.^[^ ^26^ ^]^ In addition, the apparent activation energy (Ea) of Li^+^ migration were 0.0208 eV for PDOL@PDA/PVDF‐HFP, 0.0214 eV for PDOL@PVDF‐HFP, and 0.0225 eV for PDOL@PP GPEs, respectively, which demonstrated the lower barrier for Li^+^ transport in the PDOL@PDA/PVDF‐HFP GPE.^[^ ^27^ ^]^ This was consistent with the higher ionic conductivity. The electrochemical stability of GPEs was measured via linear sweep voltammetry (LSV) (Figure 3c). The oxidation current had no significant changes below 4.3 V (vs Li^+^/Li), originating from the antioxidation ability of the PDOL GPE. The oxidation voltage of PDOL@PDA/PVDF‐HFP reached 4.57 V, which fully meted the requirement of LiFePO_4_//Li battery. This high electrochemical stabilization window was due to the hydrogen bond interactions between PDOL and PDA. The functional groups (e.g., —NH— and —OH) in PDA formed cross‐linking networks with polar oxygen atom of PDOL molecular through hydrogen bonding (Figure 1), which improved electrochemical stability of PDOL@PDA/PVDF‐HFP GPEs.^[^ ^24^, ^28^ ^]^ Besides, the *t* ^+^ values were calculated via Equation (S4) (Supporting Information) and chronoamperometry curves in Figure 3d,e,f, 0.59 for PDOL@PDA/PVDF‐HFP, higher than other GPEs (0.55, PDOL@PVDF‐HFP; 0.44, PDOL@PP) and commercial liquid electrolytes (≈0.31) at 25 °C. The high *t* ^+^ effectively can reduce the concentration polarization of Li^+^, inhibit the formation of lithium dendrites and realize a rapid rate of charging/discharging. The improved the conduction and transference of Li^+^ were attributed to much free Li^+^ resulted from that the —NH_2_ and —NH— groups in PDA interacted with the N of TFSI^−^ and the O in PDOL corresponding to results of FTIR and MD simulations.

In the electrochemical impedance spectroscopy (EIS) of the symmetric Li//Li cell, PDOL@PDA/PVDF‐HFP GPE had the lowest interface resistance *R*_i_ (39 Ω) (Figure S6d, Supporting Information) due to its good wettability, close contact between precursor and electrode. Simultaneously, the PDOL@PDA/PVDF‐HFP also represented the lowest charge transfer resistance *R*_ct_ (41.4 Ω) than the other two GPEs (58.7 Ω for PDOL@PVDF‐HFP and 68.2 Ω for PDOL@PP) (Figure S8a, Supporting Information), indicating the excellent interface contact.^[^ ^29^ ^]^ In contrast, the resistance of ex situ electrolyte was over thousands (Figure S8b, Supporting Information) and cannot operate normally at room temperature.

### 2.4 Long‐Term Cycling and Rate Performance of PDOL‐Based GPEs

**Figure 4:**
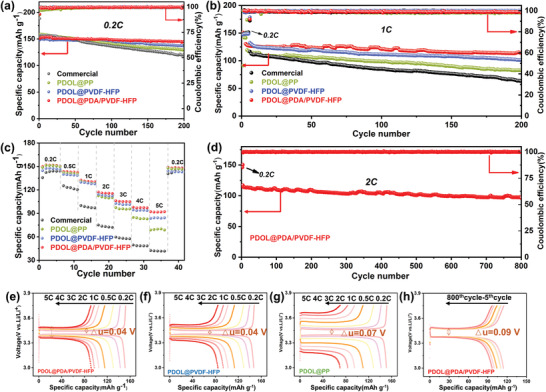
a,b,d) Cycling performance and c) Rate capability of batteries with PDOL@PP, PDOL@PVDF‐HFP, PDOL@PDA/PVDF‐HFP GPEs. and commercial electrolyte at 25 °C. e,f,g,h) Charge/discharge voltage profiles of LiFePO_4_//Li batteries with the above prepared GPEs at different current density and PDOL@PDA/PVDF‐HFP cell at 2 C at different cycles.

These splendid electrochemical properties provided a foundation for the cycling and rate performance of batteries. LiFePO_4_//Li batteries were assembled and measured during the range of 3–3.75 V at 25 °C as shown in **Figure** **4**. Visibly, after 200 cycles at 0.2 C (Figure 4a), the capacity retention of batteries with PDOL@PDA/PVDF‐HFP still remained above 96.03%, the coulomb efficiency was around 99.62%, and the discharge specific capacity was 144.6 mAh g^−1^. Meanwhile, the capacity retention was 87.13%, higher than the others (76.25%, 69.96%) after 200 cycles at 1C (Figure 4b). As the current density increased (Figure 4c), the PDOL@PDA/PVDF‐HFP battery demonstrated the highest specific capacity, apart from the competitive discharge capacity (151.6 mAh g^−1^) at 0.2 C. When the current density over 1 C, PDOL@PDA/PVDF‐HFP showed the highest specific capacity and lowest polarization voltage among them (Figure 4e–h). Especially, both PDOL@PDA/PVDF‐HFP and PDOL@PVDF‐HFP batteries showed small polarization voltages of 0.04 V at 0.2 C. Though the current density reached to 2 C, it still cycled stably over 800 cycles with a coulombic efficiency of 99.4% and few increases in polarization voltage, manifesting a low‐capacity decay of 0.021% per cycle in Figure 4d,h. Notably, that is superior to the battery performance reported in many other recent articles (Table S2, Supporting Information). It was worth mentioning that there was no overcharging or fluctuations of voltage during the cycling of NCM811//Li battery with PDOL@PDA/PVDF‐HFP, which suggested that PDOL@PDA/PVDF‐HFP had a reliable application prospect in the high‐voltage batteries (Figure S11, Supporting Information). Additionally, from the SEM cross‐sectional image of PDOL@PDA/PVDF‐HFP GPE connected with LiFePO_4_ cathode (Figure S9, Supporting Information), it was found that PDOL not only filled the nanofiber membrane uniformly forming a homogeneous GPE, but also filled the gaps between the electrodes and the electrolyte, and the holes in the cathode, which echoed the previous SEM results. And the cathode was tightly connected, which shortened the transmission path of Li^+^ ions, thus resulting in excellent electrochemical and cycling performance.

### 2.5 Suppression Performance of Lithium Dendrites

**Figure 5:**
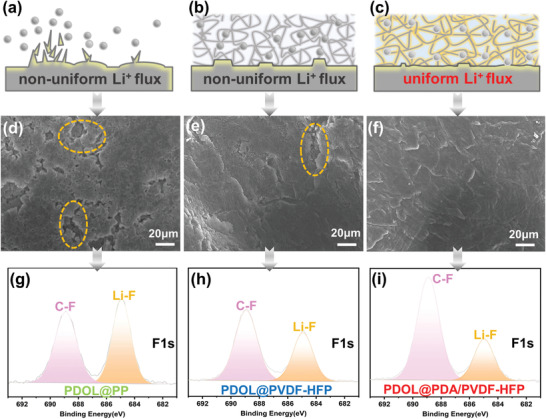
Schematic illustrations of the electrochemical plating behaviors of Li metals with three GPEs, SEM images, and F1s XPS spectroscopy of corresponding Li metals surface disassembled from batteries after cycling: a,d,g) PDOL@PP battery; b,e,h) PDOL@PVDF‐HFP battery, and c,f,i) PDOL@PDA/PVDF‐HFP battery.

In order to verify the dendrite‐suppressing ability in depth, we disassembled the batteries after 60 cycles at 0.2 C to observe the surface morphology of GPEs and Li metals extracted from batteries (**Figure** **5**). It was found that the Li surface from PDOL@PP batteries looked rough and many large fractures appeared due to unstable and uneven Li^+^ deposition as illustrated in Figure 5a,d. Accordingly, lots of protrusions were observed on the adjacent GPEs derived from accumulated dendritic Li (Figure S13, Supporting Information).

Li surface of PDOL@PVDF‐HFP cells was flatter than that of PDOL@PP, but a few wrinkles still existed on PDOL@PVDF‐HFP GPE on account of its shrinkage and curls after the precursor added (Figure 5b,e). In contrast, Li metal surface from PDOL@PDA/PVDF‐HFP cells was the smoothest without cracks (Figure 5c,f). Notably, the corresponding electrolyte surface was also the cleanest and smoothest (Figure S13c, Supporting Information). The results also reflected the difference in their cycle performance. These can be comprehended through the schematic diagram of Li deposition and trapping in Figures 5 and 1a. PDA contains abundant —OH and —NH_2_ groups, which can form good hydrogen‐bond interaction with ether groups on PDOL, improving the mechanical strength through chemical interactions. Furthermore, PDOL and PDA can interact with Li^+^ to promote the dissociation and movement of Li^+^, leading to higher conductivity and *t* ^+^ number and avoiding local Li^+^ concentration gradients.^[^ ^20^ ^]^ PDA/PVDF‐HFP membranes had higher porosity and better affinity and wettability to the precursor, which was convenient for rapid and even infiltration of mass precursors, thus making the electrolyte surface more smoothy. Therefore, the PDA@PVDF‐HFP GPE performed outstanding suppression of lithium dendrites and excellent cycling performance.

The XPS was applied to explore the chemical composition of the solid electrolyte interphase (SEI) on Li metal harvested from the LiFePO_4_//Li batteries after 60 cycles at 0.2 C as presented in Figure 5; and Figure S14 (Supporting Information). The F1s spectra exhibited two obvious peaks at around 688.9 and 684.9 eV, which were attributed to the C—F bond and LiF,^[^ ^30^ ^]^ respectively. Specifically, an appropriate intensity of LiF peak was detected on the SEI of PDOL@PDA/PVDF‐HFP battery (Figure 5i). However, the corresponding peaks of PDOL@PP or PDOL@PVDF‐HFP batteries were stronger (Figure 5g,h). Therefore, introduction of PDA effectively alleviated the decomposition of LiTFSI, reducing the content of inorganic components (LiF) with low ion conductivity in the SEI. Appropriate content of LiF can strengthen and stabilize the SEI layer,^[^ ^31^ ^]^ while too much LiF will increase battery impedance and polarization.^[^ ^32^ ^]^ Meanwhile, in the N1s and O1s spectra (Figure S14b, Supporting Information), Li_3_N (399.5 eV), —NSO_2_CF_3_ (400.6 eV), LiCO_3_ and LiOH (531.9 eV), and Li_2_O (533.4 eV) peaks were detected due to the decomposition of lithium salts,^[^ ^33^ ^]^ where Li_2_O reacted with air forming Li_2_CO_3_ and LiOH.^[^ ^30^ ^]^ Furthermore, compared with the other two samples, the larger LiN peak and Li_2_O peak were found in PDOL@PDA‐PVDF‐HFP batteries, which were conductive to Li^+^ conductivity and stability of SEI. Besides, the higher LiOH peak appeared in PDOL@PP and PDOL@PVDF‐HFP batteries, which induced the unstable SEI.^[^ ^30^ ^]^ The C1s was deconvoluted into four peaks at 284.8, 286.6, 288.5, and 289.8 eV, which ascribed to C—C/C—H, C—O—C, —CF_2_/C—N, and C—C—O/C—O bonds,^[^ ^34^ ^]^ respectively. Typically, the peaks at 286.6 and 289.8 eV appeared due to the formation of PDOL,^[^ ^31^ ^]^ and the peak at 288.5 eV corresponded to the interaction of PDA and PVDF‐HFP with Li metal. Finally, the SEI layer in PDOL@PDA/PVDF‐HFP contained appropriate amounts of rigid inorganic components for reinforcement,^[^ ^35^ ^]^ and flexible organic components, which supplied elasticity and flexibility to accommodate the volume changes during the cycling. As a result, Li dendrite growth and molecules decomposition were effectively inhibited and cycling performance was greatly enhanced.

**Figure 6:**
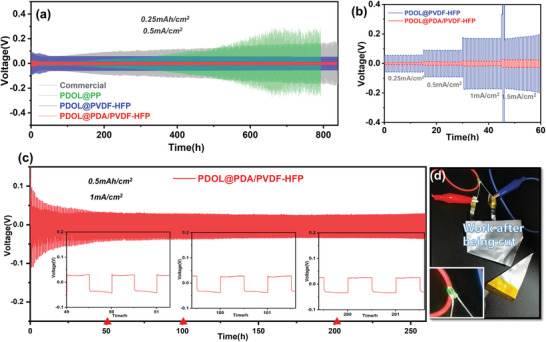
Long‐term cycling and rate performance of symmetrical cells with commercial electrolyte and the as‐prepared GPEs: long cycling at a) 1 mA cm^−2^; c) 2 mA cm^−2^ (The insets were amplified voltage curves at three time periods). b) Rate performance. d) The LiFePO_4_//Li pouch cell with PDOL@PDA/PVDF‐HFP GPE lighting a LED lamp after being cut.

The interfacial stability between electrolyte and anode is critical to the stable operation of batteries. Hence, to investigate the deposition behavior of Li^+^, the cycling performances of Li symmetric cells under different current densities were measured shown in **Figure** **6**. At a current density of 0.5 mA cm^−2^ (Figure 6a), the overpotential of cells with PDOL@PP and commercial liquid electrolyte gradually increased at 200 and 100 h, respectively, due to the continuous generation of dendrites and dead lithium during plating and striping.^[^ ^36^ ^]^ Fortunately, after PDOL was combined with porous nanofiber membranes, the overpotential remained stable and low without significant increases after 800 h. Moreover, PDOL@PDA/PVDF‐HFP cell still possessed the lower polarization at different current density, indicating its better compatibility and stability with Li metal compared with PDOL@PVDF‐HFP cell (Figure 6b). Hence, PDOL@PDA/PVDF‐HFP cell worked stably for hundred hours under 2 mA cm^−2^ (Figure 6c), ignoring the risen potential at the beginning for electrode activation and SEI formation. And the subsequent overpotential was always less than 25 mV over 250 h, showing excellent suppression of Li dendrites. Meanwhile, in order to visually evaluate the safety of PDOL@PDA/PVDF‐HFP, LiFePO_4_//Li soft pouch cell was prepared to light a light‐emitting diode (LED) lamp (Figure 6d; and Figure S15, Supporting Information). The pouch cell was normal in operation after being bent and even cut, which indicated the outstanding safety performance of PDOL@PDA/PVDF‐HFP.

## 3 Conclusion

In this study, a new flexible and safe polymer electrolyte with 3D structure was designed via combining the in situ polymerized PDOL with nanofibrous membrane. The modified porous membrane not only provided sufficient polymerization spaces for DOL, but also possessed better affinity and wettability to precursor than PP membrane, which facilitated the interweaving of PDOL and nanofibers to form a homogeneous electrolyte. Moreover, PDA can strengthen PVDF‐HFP membrane and regulate Li^+^ ion deposition. The polar groups on PDA can form hydrogen bonds with the ether oxygen group and terminal hydroxyl group on PDOL, which was beneficial to the mechanical strength and high ionic conductivity. Simultaneously, it can prevent the side reaction between the terminal hydroxyl group and Li metal, which was helpful for the electrochemical stability. Therefore, the PDOL@PDA/PVDF‐HFP electrolyte possessed high ionic conductivity of 2.39 × 10^−3^ S cm^−1^ at 25 °C, wide electrochemical window of 4.57 V (vs Li/Li), the t^+^ number of 0.59 and the lowest interface resistance. Besides, the Li symmetrical cells cycled stably for over 800 h with a tiny overpotential of 0.02 V at 1 mA cm^−2^. Furthermore, the specific capacity, coulombic efficiency and capacity retention ratios were 144.6 mAh g^−1^, 99.62% and 96.03%, respectively, after 200 cycles at 0.2 C. More importantly, the battery demonstrated an elongated cycling life with a capacity decrement of 0.021% per cycle at 2 C. This work manifested a facile method and successful design for the flexible and safe 3D GPE, which was formed via in situ polymerization of PDOL interwoven with nanofiber networks.

## 4 Experimental Section

All experimental details are shown in the Supporting Information.

## Conflict of Interest

The authors declare no conflict of interest.

## Supporting information

Supporting information

## Data Availability

The data that support the findings of this study are available on request from the corresponding author. The data are not publicly available due to privacy or ethical restrictions.

## References

[1] a) J. Holoubek , H. Liu , Z. Wu , Y. Yin , X. Xing , G. Cai , S. Yu , H. Zhou , T. A. Pascal , Z. Chen , P. Liu , Nat. Energy 2021, 6, 303; 10.1038/s41560-021-00783-z PMC7954221 33717504

[2] a) Q. Zhao , S. Stalin , C.‐Z. Zhao , L. A. Archer , Nat. Rev. Mater. 2020, 5, 229;

[3] H. Zhang , C. Li , M. Piszcz , E. Coya , T. Rojo , L. M. Rodriguez‐Martinez , M. Armand , Z. J. C. S. R. Zhou , Chem. Soc. Rev. 2017, 46, 797. 28098280 10.1039/c6cs00491a

[4] a) Y. Zhao , L. Wang , Y. Zhou , Z. Liang , N. Tavajohi , B. Li , T. Li , Adv. Sci. 2021, 8, 2003675; 10.1002/advs.202003675 PMC8025011 33854893

[5] M. Zhu , J. Wu , Y. Wang , M. Song , L. Long , S. H. Siyal , X. Yang , G. Sui , J. Energy Chem. 2019, 37, 126.

[6] a) M. Liu , S. Zhang , G. Li , C. Wang , B. Li , M. Li , Y. Wang , H. Ming , Y. Wen , J. Qiu , J. Chen , P. Zhao , J. Power Sources 2021, 484, 229235;

[7] C. H. Tsao , Y. H. Hsiao , C. H. Hsu , P. L. Kuo , ACS Appl. Mater. Interfaces 2016, 8, 15216. 27247991 10.1021/acsami.6b02345

[8] Y. Liu , C. Ding , P. Xie , X. Yan , M. Feng , Y. Liu , C. Liu , Y. Yu , Y. Lin , Mater. Chem. Front. 2021, 5, 3216.

[9] a) X. Shen , C. Li , C. Shi , C. Yang , L. Deng , W. Zhang , L. Peng , J. Dai , D. Wu , P. Zhang , J. Zhao , Appl. Surf. Sci. 2018, 441, 165;

[10] P. Xu , H. Chen , X. Zhou , H. Xiang , J. Membr. Sci. 2021, 617, 118660.

[11] D. D. Han , Z. Y. Wang , G. L. Pan , X. P. Gao , ACS Appl. Mater. Interfaces 2019, 11, 18427. 31063353 10.1021/acsami.9b03682

[12] a) J. Xiang , Y. Zhang , B. Zhang , L. Yuan , X. Liu , Z. Cheng , Y. Yang , X. Zhang , Z. Li , Y. Shen , J. Jiang , Y. Huang , Energy Environ. Sci. 2021, 14, 3510;

[13] a) F. Q. Liu , W. P. Wang , Y. X. Yin , S. F. Zhang , J. L. Shi , L. Wang , X. D. Zhang , Y. Zheng , J. J. Zhou , L. Li , Y. G. Guo , Sci. Adv. 2018, 4, eaat5383; 30310867 10.1126/sciadv.aat5383 PMC6173527

[14] J. Chen , Z. Yang , G. Liu , C. Li , J. Yi , M. Fan , H. Tan , Z. Lu , C. Yang , Energy Storage Mater. 2020, 25, 305.

[15] Q. Liu , B. Cai , S. Li , Q. Yu , F. Lv , F. Kang , Q. Wang , B. Li , J. Mater. Chem. A 2020, 8, 7197.

[16] Q. Ma , J. Yue , M. Fan , S. J. Tan , J. Zhang , W. P. Wang , Y. Liu , Y. F. Tian , Q. Xu , Y. X. Yin , Y. You , A. Luo , S. Xin , X. W. Wu , Y. G. Guo , Angew. Chem., Int. Ed. 2021, 60, 16554. 10.1002/anie.202103850 33955135

[17] T. L. F. Liu , Y. Yang , J. Yan , N. Li , J. Xue , H. Huo , J. Zhou , L. Li , Macromol. Rapid Commun. 2020, 41, 2000047. 10.1002/marc.202000047 32249484

[18] Y. Gao , X. Sang , Y. Chen , Y. Li , B. Liu , J. Sheng , Y. Feng , L. Li , H. Liu , X. Wang , C. Kuang , Y. Zhai , J. Mater. Sci. 2019, 55, 3549.

[19] H. Qiu , Z. Yang , M. Köhler , J. Ling , F. H. Schacher , Macromolecules 2019, 52, 3359.

[20] Q. Zhou , J. Ma , S. Dong , X. Li , G. Cui , Adv. Mater. 2019, 31, 1902029. 10.1002/adma.201902029 31441147

[21] J. Zhou , T. Qian , J. Liu , M. Wang , L. Zhang , C. Yan , Nano Lett. 2019, 19, 3066. 30951633 10.1021/acs.nanolett.9b00450

[22] H. A. Lee , Y. Ma , F. Zhou , S. Hong , H. Lee , Acc Chem. Res. 2019, 52, 704. 30835432 10.1021/acs.accounts.8b00583

[23] H. Haghkhah , B. Ghalami Choobar , S. Amjad‐Iranagh , J. Mol. Model. 2020, 26, 220. 32740770 10.1007/s00894-020-04464-8

[24] a) M. H. Ryou , Y. M. Lee , J. K. Park , J. W. Choi , Adv. Mater. 2011, 23, 3066; 21608049 10.1002/adma.201100303

[25] D.‐D. Han , S. Liu , Y.‐T. Liu , Z. Zhang , G.‐R. Li , X.‐P. Gao , J. Mater. Chem. A 2018, 6, 18627.

[26] X. X. Zeng , Y. X. Yin , N. W. Li , W. C. Du , Y. G. Guo , L. J. Wan , J. Am. Chem. Soc. 2016, 138, 15825. 27960330 10.1021/jacs.6b10088

[27] H. Wu , H. Jia , C. Wang , J. G. Zhang , W. Xu , Adv. Energy Mater. 2020, 11, 2003092.

[28] a) C. Ding , X. Fu , H. Li , J. Yang , J. L. Lan , Y. Yu , W. H. Zhong , X. Yang , Adv. Funct. Mater. 2019, 29, 1904547;

[29] Y. Yang , W. Wang , L. Li , B. Li , J. Zhang , J. Mater. Chem. A 2020, 8, 3692.

[30] P. N. Didwal , Y. N. Singhbabu , R. Verma , B.‐J. Sung , G.‐H. Lee , J.‐S. Lee , D. R. Chang , C.‐J. Park , Energy Storage Mater. 2021, 37, 476.

[31] Y. Gao , Z. Yan , J. L. Gray , X. He , D. Wang , T. Chen , Q. Huang , Y. C. Li , H. Wang , S. H. Kim , T. E. Mallouk , D. Wang , Nat. Mater. 2019, 18, 384. 30858569 10.1038/s41563-019-0305-8

[32] X. Ji , S. Hou , P. Wang , X. He , N. Piao , J. Chen , X. Fan , C. Wang , Adv. Mater. 2020, 32, 2002741. 10.1002/adma.202002741 33035375

[33] H. Wang , Q. Wang , X. Cao , Y. He , K. Wu , J. Yang , H. Zhou , W. Liu , X. Sun , Adv. Mater. 2020, 32, 2001259. 10.1002/adma.202001259 32734684

[34] C. Z. Zhao , Q. Zhao , X. Liu , J. Zheng , S. Stalin , Q. Zhang , L. A. Archer , Adv. Mater. 2020, 32, 1905629. 10.1002/adma.201905629 32053238

[35] S.‐M. Xu , H. Duan , J.‐L. Shi , T.‐T. Zuo , X.‐C. Hu , S.‐Y. Lang , M. Yan , J.‐Y. Liang , Y.‐G. Yang , Q.‐H. Kong , X. Zhang , Y.‐G. Guo , Nano Res. 2020, 13, 430.

[36] C. Wang , A. Wang , L. Ren , X. Guan , D. Wang , A. Dong , C. Zhang , G. Li , J. Luo , Adv. Funct. Mater. 2019, 29, 1905940.

