# Supplementary material for: Self‐Enhancing Gel Polymer Electrolyte by In Situ Construction for Enabling Safe Lithium Metal Battery
Source: Adv Sci (Weinh). 2021 Dec 11;9(4):2103663. doi: 10.1002/advs.202103663 (PMC8811824; doi:10.1002/advs.202103663)
Supplement: Supplementary file 1 — Supporting information [file ADVS-9-2103663-s001.pdf]

© 2021 Wiley-VCH GmbH

ADVANCED SCIENCE
Open Access

Supporting Information

for *Adv. Sci.*, DOI: 10.1002/advs.202103663

Self-enhancing gel polymer electrolyte by in-situ construction for enabling safe lithium metal battery

*Dongli Chen[1], Ming Zhu[2], Peibing Kang[1], Tao Zhu[1], Haocheng Yuan[1], Jinle Lan[1], Xiaoping Yang[1], Gang Sui[1]**

Supporting Information

Self-enhancing gel polymer electrolyte by in-situ construction for enabling safe lithium metal battery

*Dongli Chen[1], Ming Zhu[2], Peibing Kang[1], Tao Zhu[1], Haocheng Yuan[1], Jinle Lan[1], Xiaoping Yang[1], Gang Sui[1]**

[1] D. Chen, P. Kang, T. Zhu, Dr. H. Yuan, Prof. G. Sui, Prof. J. Lan, Prof. X. Yang

State Key Laboratory of Organic-Inorganic Composites, College of Materials Science and Engineering, Beijing University of Chemical Technology

Beijing China 100029,

Email: suigang@mail.buct.edu.cn

Dr. M. Zhu

[2] Shanghai Institute of Space Power-Sources

Shanghai China 200245,

4.1 Experimental materials

The materials used in this study including DOL (Sigma-aldrich; anhydrous contains ~75 ppm bht as inhibitor 99.8 %), $Al(OTf)_3$ (Alfa Aesar; 99 %), LiTFSI (TCI; 98 %), DMF (Aladdin; AR), acetone (Aladdin; AR), Dopamine hydrochloride (Aladdin; 98 %), PP separator (Celgard 2400), EC/DMC (1:1 by volume) with 1 M $LiPF_6$ and lithium foil (battery level) were purchased from Beijing Chemical Reagent Research Institute Co., Ltd.; The above materials were used directly without further treatment. Carbon black (Super-P) (Innochem; > 99 %), $LiFePO_4$ (battery level) and PVDF-HFP (Aldrich; Mw=455000) were dried under vacuum at 80 ℃ overnight.

4.2 The preparation of PDA/PVDF-HFP electrospinning skeleton material

The 2.1 g PVDF-HFP particles were dissolved in 10 g of a mixed solution of DMF and acetone (1:1 wt%), the solution above then was magnetically stirred at 60 °C for 8 hours to obtain a transparent and uniform spinning solution. The concentration of PVDF-HFP was about 17.4 wt%. The prepared solution was transferred into a 10 ml syringe with No. 18 (inner diameter 0.9 mm) stainless steel spinning needle, aluminum foil as the receiver.[1] The detail parameters of electrospinning were set as: the distance between the roller and needle was about 15 cm and the voltage was about 17 kV. The roller speed was about 600 r/min, and 8-9 ml solution was spun at a speed of 1.5 ml/h. The final PVDF-HFP based electrospinning membrane was dried in a vacuum oven at 80 °C for 24 hours with a thickness of 90 μm. 200 mg DA were dissolved in 100 ml Tris

(Ph=8.5, water: ethanol=1:1 volume ratio) to obtain a clear and transparent solution, and the PVDF-HFP membrane was impregnated into it at room temperature for two days.[2] The nanofiber porous membrane modified by PDA (PDA/PVDF-HFP) was successfully prepared after washed for several times with deionized water and transferred to a vacuum oven at 80 °C for 24 hours. The content of PDA in membranes was controlled about 0.17 mg cm^{-2}.

4.3 Preparation of the cathode

A certain amount of $LiFePO_4$ and Super p (the mass ratio was 8:1) were first ground for 30 minutes, then 3.5 wt% PVDF solution was added, continuing to grind for another 30 minutes to obtain a uniformly mixed slurry. The mass ratio of $LiFePO_4$: Super p: PVDF was 8:1:1.[3] Final slurry was coated evenly on the aluminum foil with a scraper (thickness of 150 μm). The electrode sheet was transferred into a vacuum oven at 80 °C for 24 h, then cut into a disc (diameter of 12 mm), and the mass loading was about 2.2 mg cm^{-2}.

4.4 Preparation of 3D gel polymer electrolyte via in-situ polymerization and $LiFePO_4$// Li battery

2 M LiTFSI were first dissolved in DOL monomer solution, magnetically stirring for 8 hours at room temperature (20 °C) to obtain a uniform and transparent precursor solution. In another screw-top bottle, 10 mg of $Al(OTf)_3$ were dissolved in 2.5 ml DOL solution, stirring magnetically at room temperature. When the initiator dissolved completely and the solution was clear and transparent. A certain amount of $Al(OTf)_3$

solution was pipetted into the LiTFSI-DOL solution, stirring for another 5 min to obtain the LiTFSI-DOL precursor solution with a certain concentration of $Al(OTf)_3$ (2 M LiTFSI and 0.4 mM $Al(OTf)_3$ initiator). All of the above steps and the batteries assembling were operated in a glove box filled with argon (O_2 < 0.01 ppm, H_2O < 0.01 ppm).

The electrolyte precursor solution (about 130 μl) was injected and in situ incorporated into as-prepared skeletons with 2025-type cells. The $LiFePO_4$//Li battery was left for 3 days to complete before testing. Similarly, batteries of referential samples including commercial IE, PDOL@PP and PDOL@PVDF-HFP were assembled. The commercial electrolyte was 1 M $LiPF_6$ in EC: DMC (1:1, v/v), and Celgard 2400 as the separator.

4.5 Materials characterization of PDOL@PDA/PVDF-HFP

4.5.1 Structure characterization

The chemical structures of DOL monomer and PDOL were analyzed by Fourier Transform Infrared spectroscopy (FTIR, Nexus 670), while other composite electrolytes were tested with ATR-FTIR (attenuated total reflection-FTIR). The measurement spectra were recorded around from 500 to 4000 cm^{-1}, scanning 128 times. Meanwhile, ^{1}H-NMR and ^{13}C-NMR spectra of PDOL were characterized by Nuclear Magnetic Resonance Spectroscopy (NMR, Bruker AVANCE III HD 400 MHz) to qualitatively analyze of the composition and structure of the sample, with tetramethylsilane (TMS) as an internal reference and DMSO-d_6 as the deuterated

solvent. The PDOL was dissolved in tetrahydrofuran (THF) solvent for Gel Permeation Chromatography (Breeze2 GPC) measurement to analyze the relative molecular mass and relative molecular weight distribution (polydispersity index) of polymer samples. Wetting performance and contact angle between the electrolyte precursor solution and framework membranes were conducted on the Contact Angle Meter (XG-CAMB).

The samples were immersed into n-butanol for 2 h to obtain the porosity (P), which was calculated based on equation 1 below:[2]

$$P\ \% = \frac{(W_2 - W_1)/\rho_n}{V} \qquad (1)$$

where W_1 and W_2 are the membrane weights before and after soaked into n-butanol, V represents the volume of membranes, ρ_n is the density of n-butanol (0.8098 g cm^{-3}).

Microscopic morphologies and microstructures of as-prepared samples were observed with scanning electron microscope (SEM, Supra55, Carl Zeiss), with scanning voltage of 10 KV. The electrolyte dried in vacuum at 60 ℃ for 6 h to evaporate the liquid components inside and Li metals removed from the battery were washed with DOL solution before observation. The crystallinity of samples was tested on Differential Scanning Calorimetry (TA DSC250) at the range of -20 °C to 60 °C with temperature change rate of 10 °C/min in an air atmosphere. The thermal stability of lithium salt and polymer was determined by the thermogravimetric analysis test (TGA, TA Q50), starting from room temperature to 700 °C under the nitrogen atmosphere with temperature rising rate of 10 °C min^{-1}. Tensile performance tests were carried on the universal tensile tester (Instron-1121) to test the mechanical properties of the

framework materials and composite electrolytes in a slender rectangle of 10 mm×50 mm. The distance between the upper and lower clamps was 25 mm, tensile rate about 5 mm min^{-1}, recording the stress and strain changes during the deformation process. X-ray photoelectron spectroscopy (XPS, 2500VB2+PC, Rigaku Corporation, Japan) measurements were carried out to explore the component of the skeletons and Li metals surface.

4.5.2 Electrochemical measurements

Electrochemical impedance spectroscopy (EIS) measurements are performed on the as-prepared composite polymer electrolyte with PGSTAT 302N electrochemical workstation. Evaluate the ionic conductivity during the frequency range of 0.1 Hz to 200 kHz at room temperature and from 25 ℃ to 90 ℃. Calculating ionic conductivity by the following formula 2 :

$$\sigma = \frac{L}{R_b S} \qquad (2)$$

where σ, L and S are the ionic conductivity, the thickness and the area of GPEs, respectively. R_b is the bulk impedance.

The calculation of reaction activation energy (Ea) follows the formula 3:[4]

$$E_a = RT \ln\frac{A}{\sigma} \qquad (3)$$

where the E_a represents the activation energy that needed for Li^+ conduction. R is the molar gas constant and T is the measurement temperature. A is the pre-exponential factor and σ represents Li^+ conductivity[4a, 5].

The Li//Li symmetric cells were measured to obtain the Li^+ transference number (t^+). The AC impedance frequency was 200 kHz-0.1 Hz. Chronoamperometry method was used to determine the initial and steady-state current ($I_{0,}$ I_S). The applied polarization voltage (△V) was 2 mV. t^+ is calculated according to the formula：[6]

$$t^{+} = \frac{I_S(\Delta V - I_0 R_0^{el})}{I_0(\Delta V - I_S R_S^{el})} \quad (4)$$

where R_0^{el} and R_s^{el} are the resistance values before and after polarization, respectively.

Meanwhile, in order to investigate the stability of the interface of Li anode against GPEs, the assembled Li symmetrical cells were cycled under different current density charging for 1 h and discharging for 1 h with the LAND (CT2001A) test system.

Series of cyclic voltammograms (CV) measurements at different scan rates of 0.1-0.5 mV s^{-1} were conducted and analyzed with Randles-Sevick equation to achieve the Li^+ ion diffusion coefficients (D_{Li}) of different GPEs. The equation is shown below:[7]

$$i_p = 2.69 \times 10^5 n^{\frac{3}{2}} A D_{Li} V^{\frac{1}{2}} \Delta C_0 \quad (5)$$

where the D_{Li} stands for the diffusion coefficient of Li^+. I_p is the peek current, and n represents the number of electrons involved in the redox. A is the area of electrode. C_{Li} represents the concentration of Li^+ and ν refers to the scanning rate.

The Li//stainless steel (SS) asymmetric cells were applied to explore the electrochemical stability via linear sweep voltammetry with the applied potential range of 2-7 V (vs. Li/Li) at the scanning rate of 5 mV s^{-1}.

The $LiFePO_4$//Li (CR2025) coin cells were used to evaluate the electrochemical characteristics. The LAND system was used to carry out charge and discharge cycling test on the cells during the potential range of 3.0 V-3.75 V. For studying the rate performance, the battery was cycled about 5 cycles under different charge and discharge current density of 0.2 C, 0.5 C, 1 C, 2 C, 3 C, 4 C, 5 C, 0.2 C.

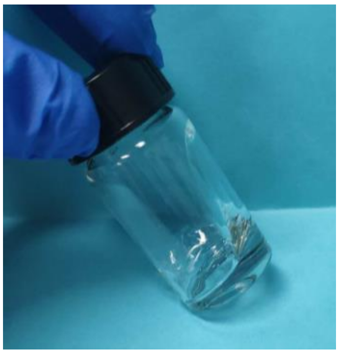

Figure S1. Optical picture after DOL polymerization in the sealed bottle.

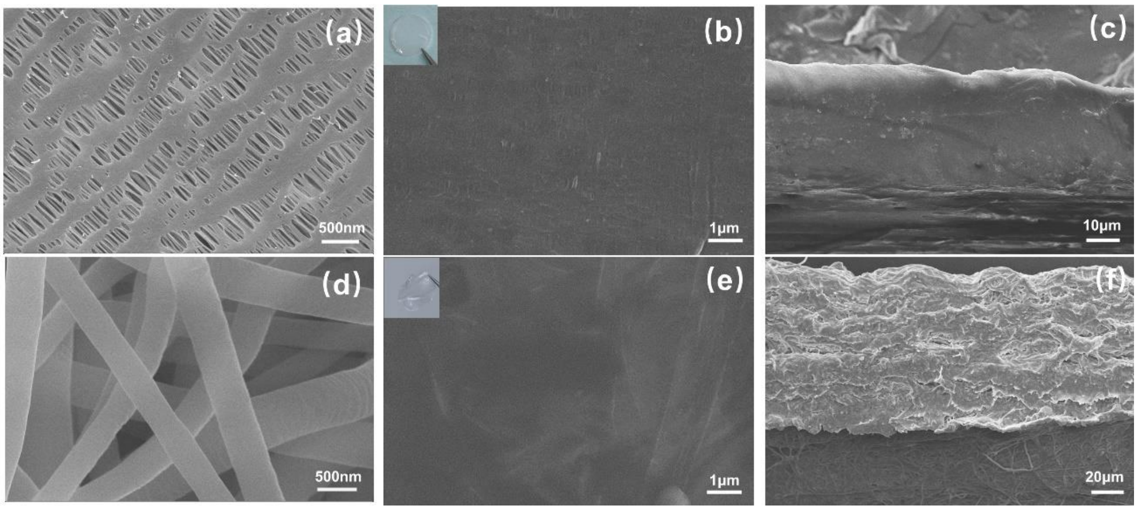

Figure S2. SEM images of membranes surface: a) PP membrane, and d) PVDF-HFP membrane; GPEs surface: b) PDOL@PP GPE, and e) PDOL@PVDF-HFP GPE, (the insets are optical images of PDOL@PP and PDOL@PVDF-HFP GPEs). GPE cross section: c) PDOL@PP GPE, and f) PDOL@PVDF-HFP GPE.

The presence of peeks (286.0 eV, 288.2 eV, 533.0 eV) in C1s and O1s belonging to the C=N bonding, and oxygen-containing C=O, C-O groups, corroborated the

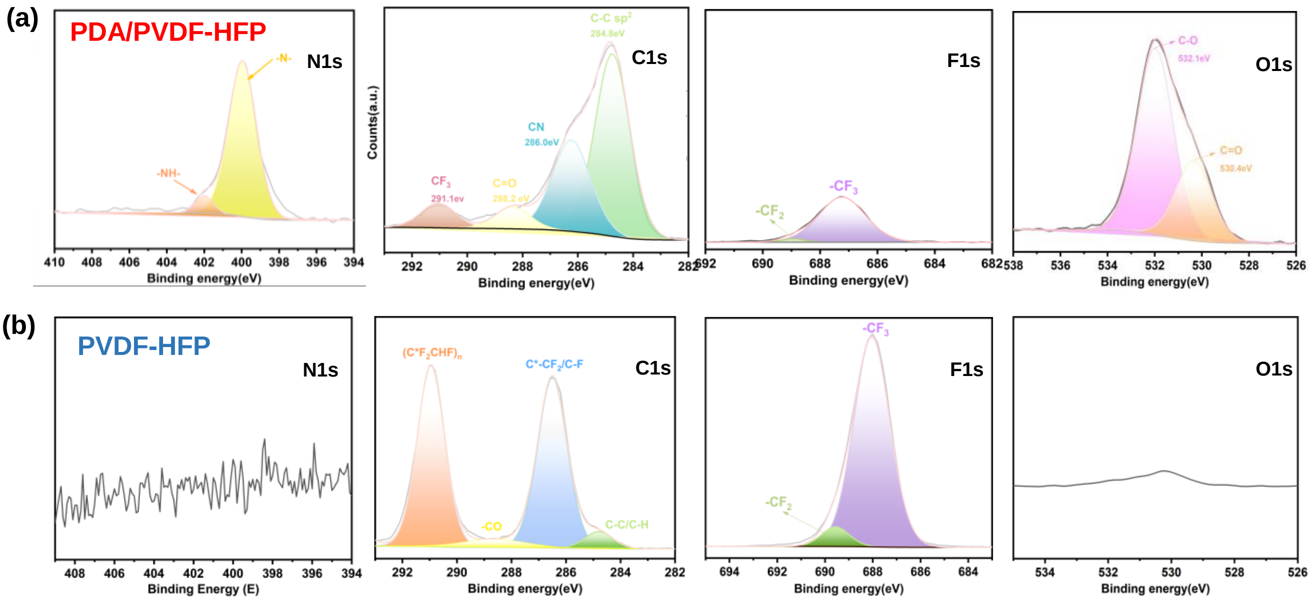

successful introduction of PDA in **Figure S3.**

Figure S3. N1s, C1s, F1s and O1s XPS spectrum of membranes: a) PDA/PVDF-HFP, b) PVDF-HFP.

The PP, PDA/PP, PVDF-HFP and PDA/PVDF-HFP membranes were treated at 150 ℃ for 0.5 h, and the results were shown in **Figure S4**. The PP membrane underwent serious shrinkage, and it was alleviated after being modified by PDA. The PVDF-HFP membrane was slightly deformed. Furthermore, the PDA@PVDF-HFP membrane showed almost no shrinkage, which featured the best thermal stability among those membranes.

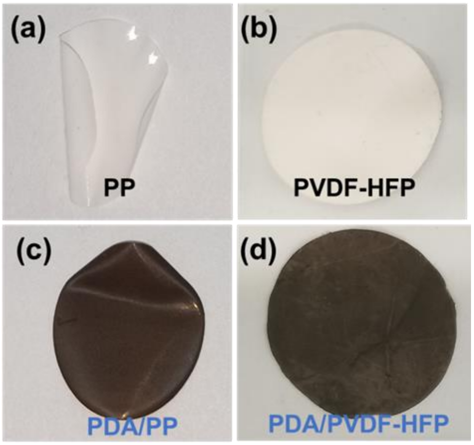

Figure S4. The thermal stability testing of different membranes at 155 ℃ for 0.5 h

The modulus of PVDF-HFP fiber membrane increased by 1.7 MPa after modified with PDA (**Table S1**). The modulus of PDOL@PDA/PVDF-HFP was further increased after PDOL was integrated owing to the outstanding interaction between PDOL and PDA. This was beneficial to inhibit the growth of lithium dendrites during cycling.

Table S1. The Young's Modulus of PVDF-HFP, PDA/PVDF-HFP, PDOL@PVDF-HFP and PDOL@PDA/PVDF-HFP through stress-strain curves.

**Samples**	**Young's Modulus (MPa)**
PVDF-HFP	14.1
PDA/PVDF-HFP	15.8
PDOL@PVDF-HFP	12.9
PDOL@PDA/PVDF-HFP	22.2

In **Figure S5a**, the porosity of PVDF-HFP and PDA/PVDF-HFP were 76.7 % and 68.3 %, respectively. The porosity was greatly reduced to 29.1 % after PP coated with PDA, presenting the low porosity. Besides, the TGA results **(Figure S5c)** also affirmed that after liquid DOL transferring into PDOL, the thermal stability of electrolyte had

been greatly enhanced. Decomposition of PDOL occurred at 151 ℃, and LiTFSI began to decompose at 400 ℃. The decomposition temperature of polymer electrolyte was over 100 ℃, satisfying the normal use of the battery.

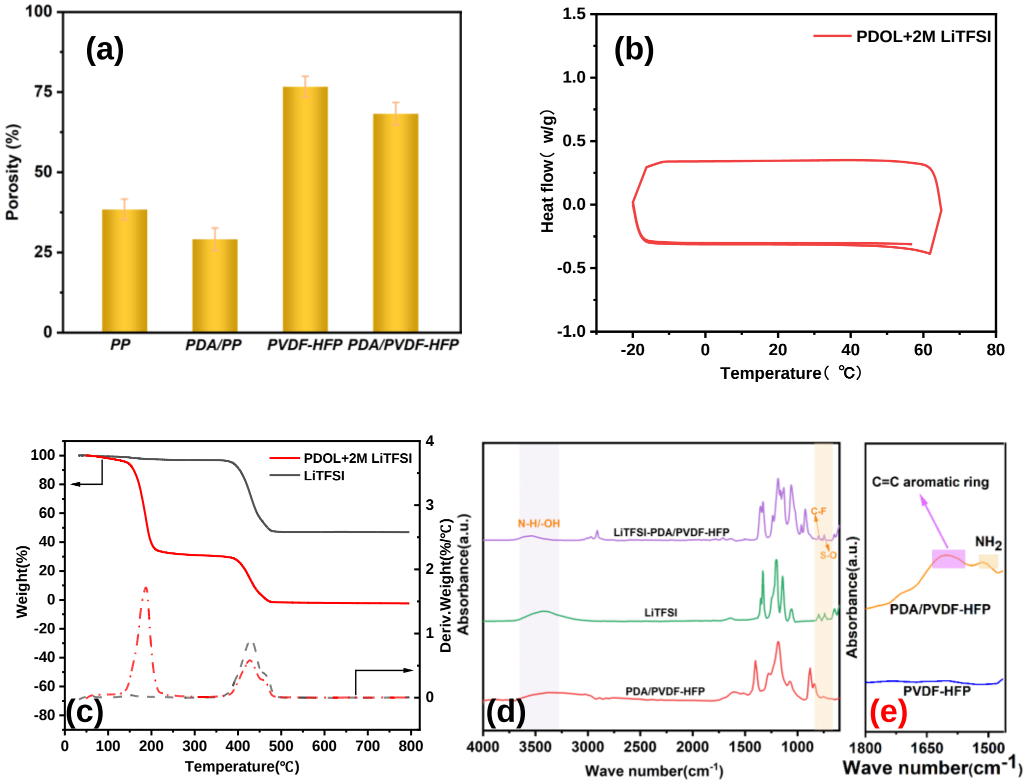

Figure S5. a) The porosity of PP, PDA/PP, PVDF-HFP, PDA/PVDF-HFP membranes; b) DSC profiles for PDOL via in-situ polymerization; c) TG curves for PDOL and LiTFSI salt; d) FTIR spectroscopy of LiTFSI salt, PDA/PVDF-HFP membrane and LiTFSI-PDA/PVDF-HFP membrane.; e) FTIR spectroscopy of PVDF-HFP and PDA/PVDF-HFP membranes at the range of 1800-1465 cm^{-1}.

MD (molecular dynamics) simulations were performed on the different systems consisting of 30 PDOL chains and 30 other chains. A PDOL chain was composed of 10 repeat units with a structure H-[O-CH_2-O-CH_2-CH_2]$_{10}$-H. The short chains made our

simulation equilibrated rapidly, and we can get some useful information in she short time. For PVDF-HFP, the typical random molecular chain was produced by Material studio's Polymer Builder. PDA's structure was not fully researched, so the most stable dimer structure suggested by Chun-Teh Chen was used in this paper.[8] The interaction in the system was described using the powerful forcefield COMPASS which was developed for the condensed-phase simulation. It had been widely used in various situation such as common organic molecules, macromolecules, and inorganic materials.

The simulations were prepared in the following way: (Ⅰ) The three-dimensional, low-density, cubic system was produced by Amorphous cell at 298 K and 1 atm. (Ⅱ) The system was equilibrated by a series of successive NVT/NPT processes under high temperature and pressure, followed by a long-time simulation at 363 K and 1 atm. (Ⅲ) The barostat of produce phase was changed to velocity rescale barostat which can produce the correct NPT statistical ensemble. The Ewald summation method was used for treatment of long-range electrostatic forces between partial charges. The cutoff for dispersion term was 12.5 Å, while a correction was made for the van der Waals term neglected beyond the cutoff. The r was distance between two atoms, and g(r) was the probability of the corresponding.[9]

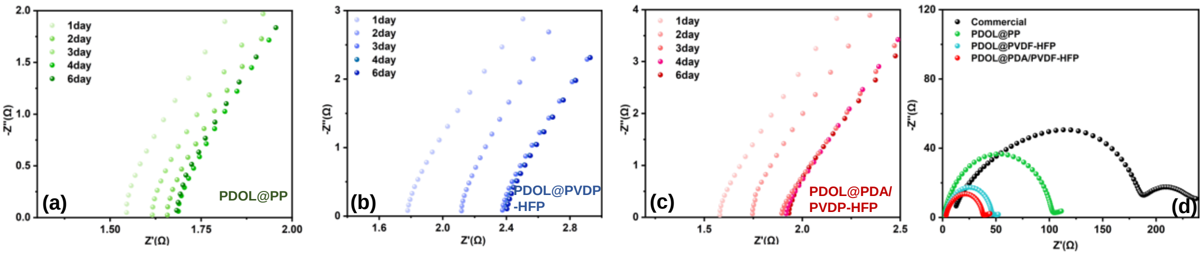

Figure S6. a-c) Nyquist plots of PDOL@PP, PDOL@PVDF-HFP and PDOL@PDA/PVDF-HFP GPEs; d) AC impedance profiles of Li//Li cells with the above GPEs and commercial liquid electrolyte.

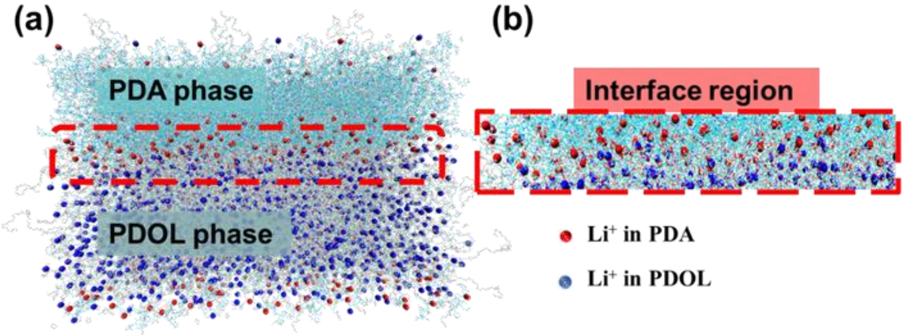

Figure S7. The a) Dissociation of Li^+ in the two-phase interface region from MD simulation and b) the enlargement of the interface region.

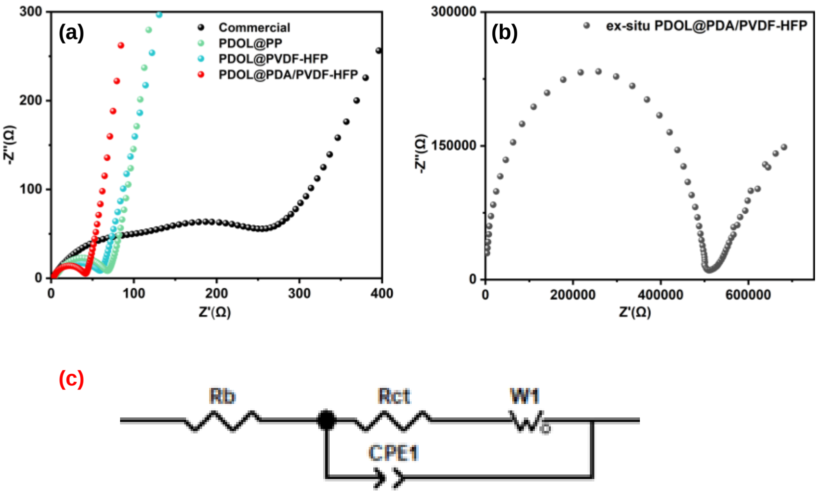

Figure S8. EIS plots of $LiFePO_4$//Li cells with a) in-situ GPEs and commercial electrolyte; b) ex-situ PDOL@PDA/PVDF-HFP GPE; c) the equivalent circuits of Nyquist plots.

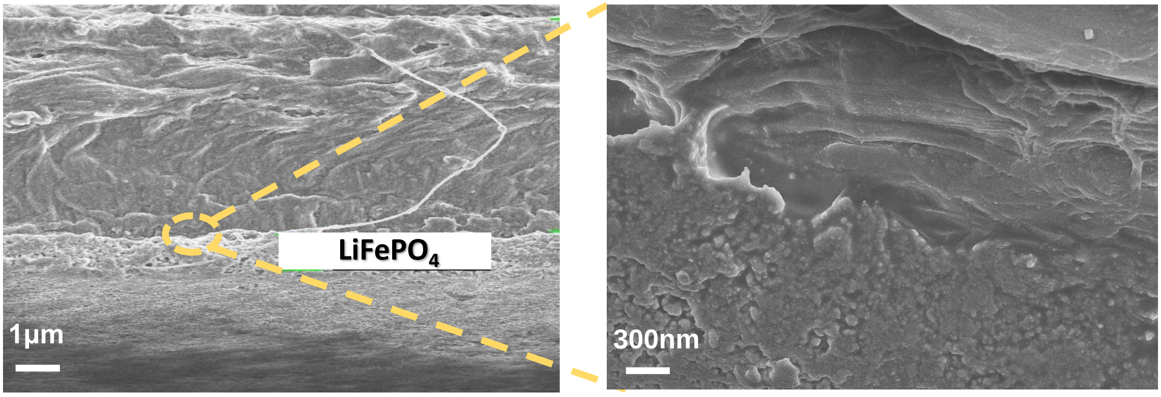

Figure S9. SEM images of cross section of $LiFePO_4$ with PDOL@PDA/PVDF-HFP and the corresponding magnified image.

To explore and understand the electrochemical reaction processes of different GPEs in deep, CV curves of LFP//Li batteries at different scan rates were analyzed as shown in **Figure S10**. At 0.1 mV s^{-1}, the potential gaps (△V) between the oxidation and reduction peaks of PDOL@PDA/PVDF-HFP and PDOL@PVDF-HFP cells were relatively close (about 0.19 V) and smaller than PDOL@PP (0.22 V) **(Figure S10a)**. Besides, the positions of the redox peaks corresponded well to the plateaus in the charge-discharge curves during the potential of 3-3.8 V **(Figure 4e)**. The redox peaks of PDOL@PDA/PVDF -HFP cell was stronger than that of PP in **Figure S10a**

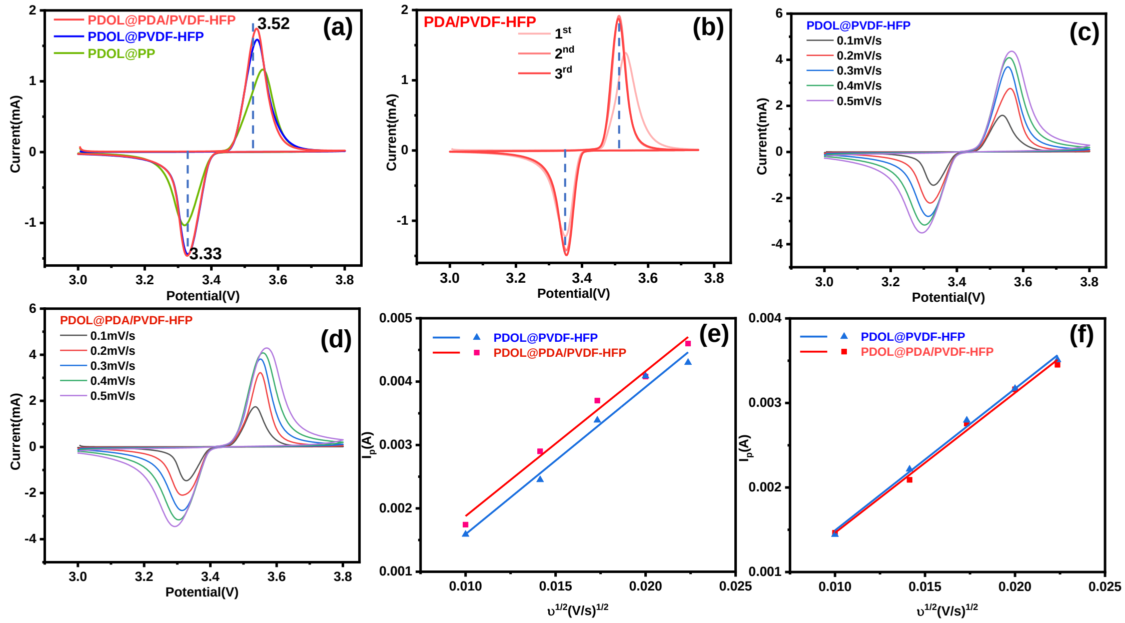

Figure S10. a) EIS plots of $LiFePO_4$//Li cells with as-prepared GPEs and commercial electrolyte; CV curves: b) PDOL@PDA/PVDF-HFP cell at 0.1 mV/s, and at different scan rates of 0.1-0.5 mV s^{-1}: c) PDOL@PVDF-HFP cell; d) PDOL@PDA/PVDF-HFP cell; CV peak current data of PDOL@PVDF-HFP and PDOL@PDA/PVDF-HFP cells: e) the anodic oxidation process and f) the cathodic reduction process versus the square root of scan rates.

indicating a faster diffusion of lithium ion. The first three CV curves overlapped well shown in **Figure S10b**, reflecting a well-reversible electrochemical redox process,[10] which was obviously beneficial to the cycle stability of batteries.[11] With the scan rate increasing, the redox peaks became wider, relating to the gradual increase in the polarization of $LiFePO_4$ but still in an obvious peak shape **(Figure S10c, S10d)**. Through the relationship between different scan rates and corresponding peak current (Ip) **(Figure S10e, f)**, the Li^+ diffusion coefficients (D_{Li}) were figured out via equation

5. The D_{Li} of PDOL@PDA/PVDF-HFP and PDOL@PVDF-HFP (~10^{-9} cm^2 s^{-1}) were higher than that of PP with commercial electrolyte (~10^{-13} cm^2 s^{-1}).[12]

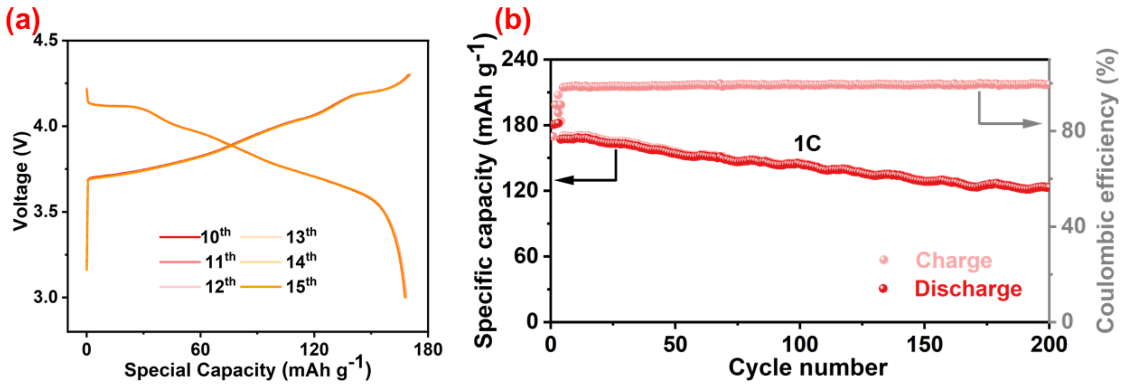

Figure S11. a) Charge/discharge voltage profiles at different cycles and b) the cycle performance of NCM811//Li battery with PDOL@PDA/PVDF-HFP at 1 C (room temperature).

The battery performance of $LiFePO_4$//Li with the PDOL@PDA/PP was conducted to expound the superiority of PDOL@PDA/PVDF-HFP (**Figure S12**). After 200 cycles at 0.2 C, the capacity retention of battery with PDOL@PDA/PP was 86.36 %, the coulomb efficiency was 99.57 %, which was higher than that of PDOL@PP (80.71 %, and 99.43 %), while it was worse than that of PDOL@PDA/PVDF-HFP (96.03 %, and 99.62 %). As the current density increased, the specific capacity of the PDOL@PDA/PP battery decreased (90.8 mAh g^{-1} at 3 C and 81.8 mAh g^{-1} at 4 C), which was lower than that of PDOL@PDA/PVDF-HFP (106.1 mAh g^{-1} at 3 C and 97.8 mAh g^{-1} at 4 C).

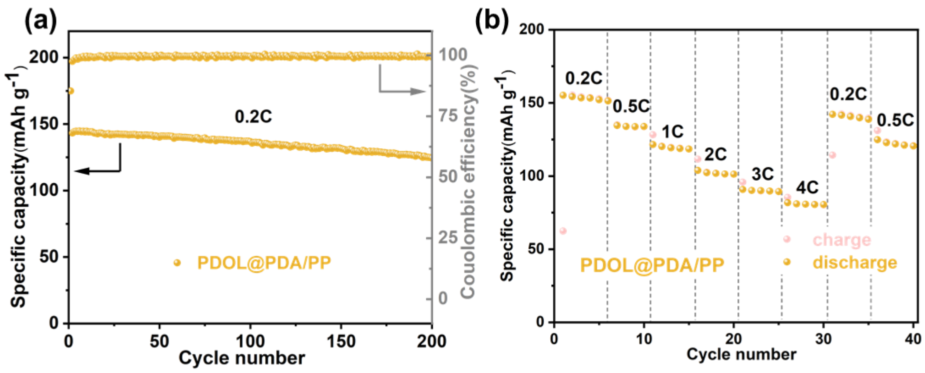

Figure S12. a) Cycling performance and c) Rate capability of $LiFePO_4$//Li battery with PDOL@PDA/PP under room temperature.

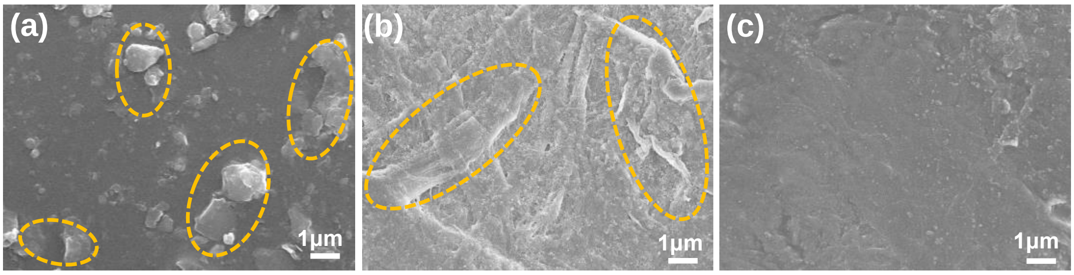

Figure S13. SEM images of a) PDOL@PP, b) PDOL@PVDF-HFP, and c) PDOL@PDA/PVDF-HFP GPEs surface after cycling.

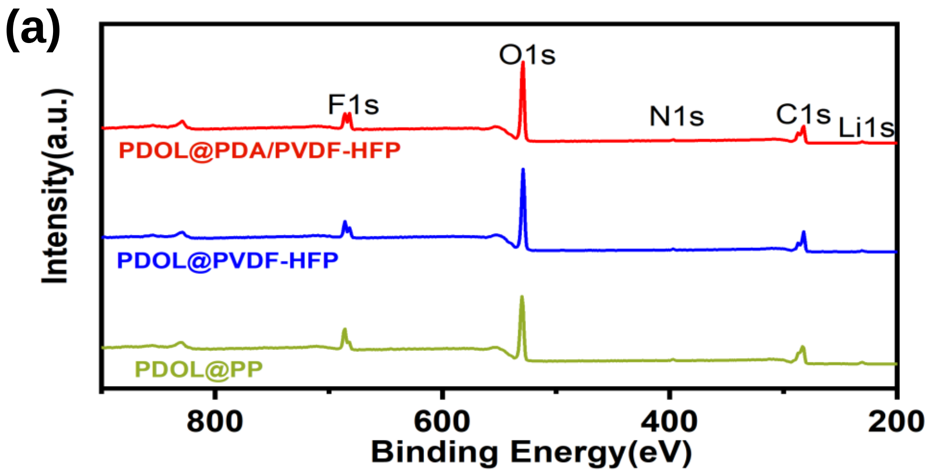

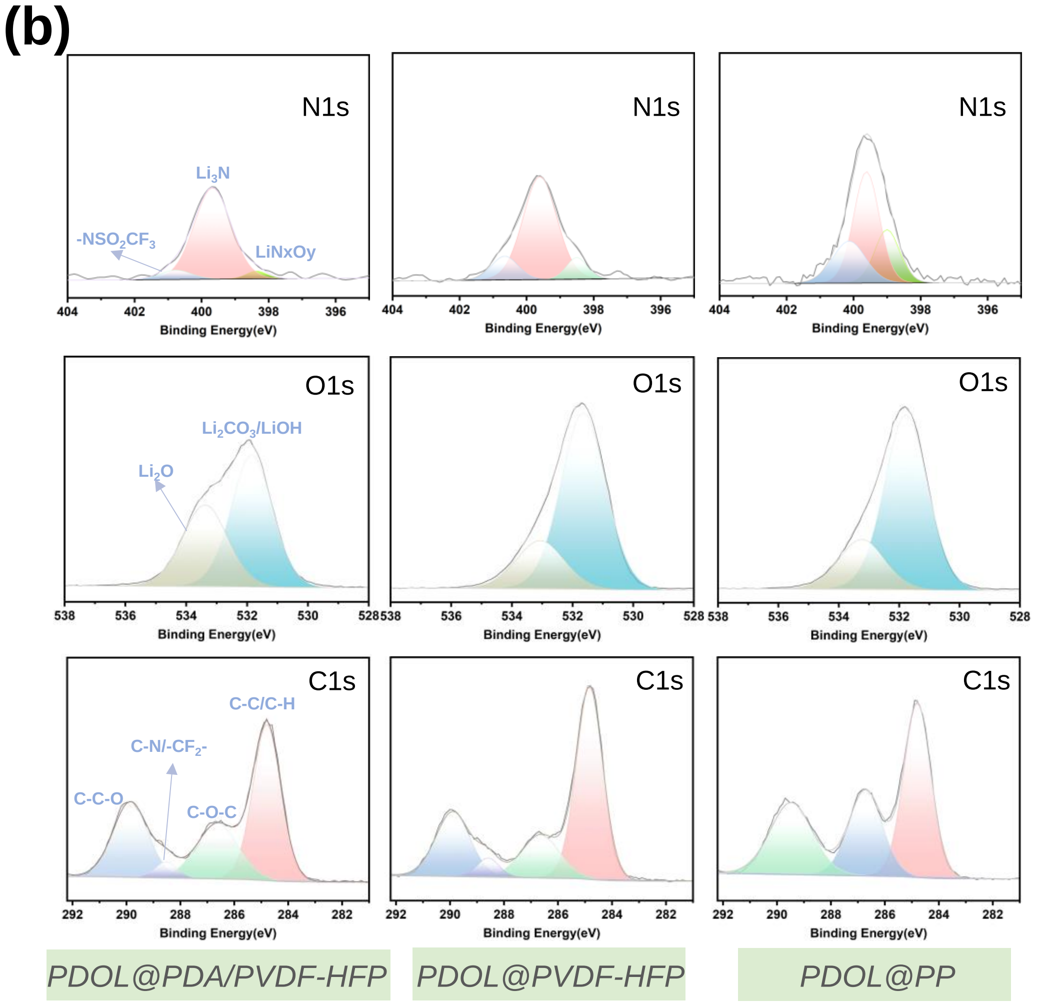

Figure S14. a) XPS spectrum of Li metal surface dissembled from the batteries with PDOL@PP, PDOL@PVDF-HFP, PDOL@PDA/PVDF-HFP GPEs; b) N1s, O1s and C1s XPS spectroscopy of the above GPEs.

The peak at 399.4 eV belonged to C-N stretching vibration.

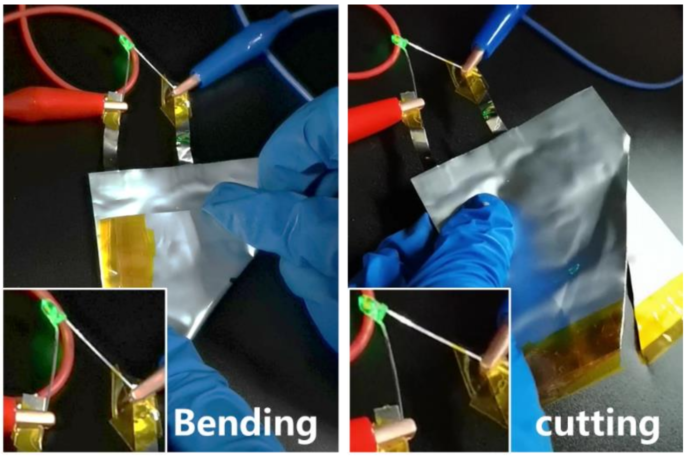

Figure S15. The $LiFePO_4$//Li pouch cell with PDOL@PDA/PVDF-HFP GPE lighting a LED lamp after being bent and cut (Inset pictures: the enlargement of lamp).

Table S2. Comparison of PDOL@PDA/PVDF-HFP electrolyte with other polymer electrolytes (PEs) via in-situ polymerization

In situ PEs	Supporting skeleton	Initiator, Temperature	Additives, solvent	Ionic transference number, conductivity	Battery performance	reference
Poly(2-(((2-oxo1,3-dioxolan-4-yl) methoxy) carbonylamino)-ethyl methacrylate)（PCUMA)	Cellulose	AIBN, 60℃	LiDFOB, 400% (the mass ratio to the monomer) Succinonitrile (SN)	t^+=0.62, 1.07 mS/cm (30 °C)	$LiCoO_2$//Li, 30℃, 0.5 C, 300 cycles, capacity retention: 88.0% (4.5 V); $LiCoO_2$//Li, 30℃, 0.5 C, 100 cycles, capacity retention: 82.4% (4.6 V);	Chem. Mater., 2020, 32 [13]
Poly(poly(ethylene glycol) methacrylate-co-methyl methacrylate)	Cellulose	ethyl α-bromophenylacetate, 70℃	LiI, 18-Crown-6-ether	t^+=0.37, 0.03 mS/cm (30 °C)	$LiFePO_4$//Li, 60℃, 0.1 C, 150 cycles, capacity retention: 82.0%	Macromolecules, 2021, 54 [14]
poly(formaldehyde)	Cellulose nonwoven	LiDFOB, 80℃	60% SN	0.114 mS/cm (RT)	$LiCoO_2$//Li, 0.3 C, 200 cycles, capacity retention :88%, capacity decay rate: 0.06%;	Adv. Sci. 2020, 7 [15]
poly(ethylene glycol dimethacrylate-1,2-ethane dithiol) (P(EGDMA-EDT))	PI nowoven	n-hexylamine	/	t^+=0.45, 0.03 mS/cm (25°C)	$LiFePO_4$//Li, 25℃, 0.2 C, 138.4 mAh/g after 100 cycles	ACS Appl. Mater. Inter., 2021, 13, 34274−34281[16]
Poly(ε-caprolactone) (PCL)	PE	$Sn(Oct)_2$, 100℃	PC	t^+=0.5; 0.021 mS/cm (27℃)	$LiFePO_4$//Li, 60℃, 0.1 C, 150 cycles, capacity retention:72.0%	Mater. Today Energy, 2021, 21 [17]
PDOL	Glass fiber	$AlI_3/LiPF_6$	/	t^+=0.62	Without CIE, NCM811, 0.2C, from ~200 mAh/g to ~120 mAh/g after 100 cycles	Adv. Funct. Mater., 2021, [18]
PDOL	PP	tris(pentafluorophenyl)	/	t^+=0.58, 1.16 mS/cm (30°C)	Li//S, 30°C, 0.2 C, from 1060 mAh/g, to 660 mAh/g	Energy Environ.

In situ PEs	Supporting skeleton	Initiator, Temperature	Additives, solvent	Ionic transference number, conductivity	Battery performance	reference
		borane			after 550 cycles, capacity decay rate: 0.094%	Sci., 2021,14 [19]
PDOL and poly(lithium allyl-sulfide)	Commercial separator	$LiPF_6$	diallyldisulfide	0.2 mS/cm (30°C)	$LiFePO_4$//Li, 30℃, 3 C, 400 cycles, capacity retention :80%, capacity decay rate: 0.05%;	Nano Lett., 2019, 9 [20]
PDOL	Glass fiber	LiDFOB, 60℃	30% SN	0.39 mS/cm (RT)	$LiFePO_4$//Li, RT, 1 C, 1000 cycles, capacity retention: 83.55%	J. Mater. Chem. A, 2020, 8 [21]
PDOL	**PDA/PVDF-HFP nonwoven**	**$Al(OTf)_3$, RT**	**/**	**t^+=0.59; 0.29 mS/cm (25℃)**	**$LiFePO_4$//Li, 20℃, 2 C, 800 cycles, capacity retention: 82.5%, capacity decay rate: 0.022%; 0.2 C, after 200 cycles, 145.4 mAh/g, capacity decay rate: 94.8%**	***This work***

Reference

[1] M. Zhu, J. Wu, B. Liu, W.-H. Zhong, J. Lan, X. Yang, G. Sui, *J. Membrane Sci.* **2019**, *588*, 117194.

[2] C. Shi, J. Dai, S. Huang, C. Li, X. Shen, P. Zhang, D. Wu, D. Sun, J. Zhao, *J.Membrane Sci.* **2016**, *518*, 168.

[3] M. Li, H. Li, J.-L. Lan, Y. Yu, Z. Du, X. Yang, *J. Mater. Chem. A* **2018**, *6*, 19094.

[4] a) K. Pan, L. Zhang, W. Qian, X. Wu, K. Dong, H. Zhang, S. Zhang, *Adv. Mater.* **2020**, *32*, e2000399. b) L. Liu, J. Mo, J. Li, J. Liu, H. Yan, J. Lyu, B. Jiang, L. Chu, M. Li, *J. Energy Chem.* **2020**, *48*, 334.

[5] S. Tang, W. Guo, Y. Fu, *Adv. Energy Mater.* **2020**, 2000802.

[6] a) J. Holoubek, H. Liu, Z. Wu, Y. Yin, X. Xing, G. Cai, S. Yu, H. Zhou, T. A. Pascal, Z. Chen, P. Liu, *Nat. Energy* **2021**, *6*, 303. b) S. Liu, Y. Zhao, X. Li, J. Yu, J. Yan, B. Ding, *Adv. Mater.* **2021**, e2008084. c) F. Zeng, Y. Sun, B. Hui, Y. Xia, Y. Zou, X. Zhang, D. Yang, *ACS Appl. Mater. Interfaces* **2020**, *12*, 43805.

[7] J. Wu, N. You, X. Li, H. Zeng, S. Li, Z. Xue, Y. Ye, X. Xie, *J. Mater. Chem. A* **2019**, *7*, 7644.

[8] C. Chen, F. J. Martin-Martinez, G. S. Jung, M. J. Buehler, *Chem. Sci.* **2017**, *8*, 1631.

[9] M. Yang, Y. Liu, A. M. Nolan, Y. Mo, *Adv. Mater.* **2021**, 33, 2008081.

[10] G. Xu, A. Kushima, J. Yuan, H. Dou, W. Xue, X. Zhang, X. Yan, J. Li, *Energy Environ. Sci.* **2017**, *10*, 2544.

[11] Pravin N. Didwal, Y. N. Singhbabu, R. Verma, B.-J. Sung, G.-H. Lee, J.-S. Lee, D. R. Chang, C.-J. Park, *Energy Storage Mater.* **2021**, *37*, 476.

[12] F. Liu, Y. Yang, J. Yan, Ning Li, J. Xue, H. Huo, J. Zhou, Lin Li, Macromol. rapid comm. **2020**, *41*, 2000047.

[13] C. Wang, S. Dong, Z. Hu, R. Hu, Z. Guo, T. Wang, G. Cui, L. Chen, Chem. Mater. **2020**, *32*, 9167−9175.

[14] L. Yu, J. Wang, H. Gan, S. Li, X. Xie, Z. Xue, Macromolecules **2021**, *54*, 874−887.

[15] H. Wu, B. Tang, X. Du, J. Zhang, X. Yu, Y. Wang, J. Ma, Q. Zhou, J. Zhao, S. Dong, G. Xu, J. Zhang, H. Xu, G. Cui, L. Chen, Adv Sci. **2020**, *7*, 2003370.

[16] R. Xu, B. Xiao, C. Xuan, S. Gao, J. Chai, S. Liu, Y. Chen, Y. Zheng, X. Cheng, Q. Guo, Z. Liu, ACS Appl. Mater. Inter. **2021**, *13*, 34274−34281.

[17] M. Sun, Z. Zeng, L. Peng, Z. Han, C. Yu, S. Cheng, J. Xie, Mater. Today Energy **2021**, *21*, 100785.

[18] Y. Chen, F. Huo, S. Chen, W. Cai, S. Zhang, Adv. Funct. Mater. **2021**, 2102347.

[19] J. Xiang, Y. Zhang, B. Zhang, L. Yuan, X. Liu, Z. Cheng, Y. Yang, X. Zhang, Z. Li, Y. Shen, J. Jiang, Y. Huang, Energy Environ. Sci. **2021**, *14*, 3510.

[20] J. Zhou, T. Qian, J. Liu, M. Wang, L. Zhang, C. Yan, Nano Lett. **2019**, *19*, 3066.

[21] Q. Liu, B. Cai, S. Li, Q. Yu, F. Lv, F. Kang, Q. Wang, B. Li, J. Mater. Chem. A **2020**, *8*, 7197.
